# Interpretable machine learning–based prediction of 90-day mortality after endovascular treatment in acute large vessel occlusion stroke

**DOI:** 10.3389/fneur.2026.1878562

**Published:** 2026-09-03

**Authors:** Yuanfang Zhou, Xiaoxia Li, Guokuan Feng, Chuanhang Lin, Bao Liao

**Affiliations:** 1 Department of Neurology, Hezhou People's Hospital, Hezhou, Guangxi, China; 2 Department of Neurology, Baise People's Hospital, Baise, Guangxi, China; 3 Youjiang Medical University for Nationalities, Baise, Guangxi, China

**Keywords:** acute ischemic stroke, explainable artificial intelligence, large vessel occlusion, machine learning, mechanical thrombectomy, prognostic model

## Abstract

**Background:**

Despite high recanalization rates after endovascular treatment (EVT) for acute ischemic stroke due to large vessel occlusion (AIS-LVO), 15%−20% of patients still die within 90 days. Existing prognostic models predominantly target functional disability rather than mortality, and rarely incorporate calibration, clinical utility, or interpretability. We aimed to develop and internally validate interpretable machine learning (ML) models for dynamic post-EVT in-hospital prediction of 90-day all-cause mortality in AIS-LVO patients with successful recanalization.

**Methods:**

We retrospectively enrolled 449 AIS-LVO patients with successful EVT (modified Thrombolysis in Cerebral Infarction grade 2b−3) at a nationally certified Advanced Stroke Center in Guangxi, southwestern China, between January 2022 and May 2025. Candidate predictors included baseline characteristics, admission laboratory indicators, procedural variables, and post-procedural complications available during hospitalization. Patients were randomly split 8:2 into training (*n* = 359) and independent test (*n* = 90) sets, stratified by 90-day mortality. Fifteen ML algorithms were optimized via Bayesian hyperparameter search with 5-fold cross-validation, and the top five underwent bootstrap optimism correction. Performance was evaluated across discrimination, calibration, clinical utility (decision curve analysis), and reclassification (NRI/IDI). SHapley Additive exPlanations (SHAP) and permutation importance were applied for interpretability.

**Results:**

The 90-day mortality rate was 19.6% (88/449). Random Forest achieved favorable overall test-set performance (AUC = 0.806; Brier score = 0.128; Hosmer–Lemeshow *p* = 0.315), with positive net benefit across threshold probabilities of 0.10–0.60. SHAP analysis identified stroke-associated pneumonia, D-dimer, age, acute renal insufficiency, fasting blood glucose, NIHSS score, brain herniation, and symptomatic intracranial hemorrhage as top contributors, concordant with permutation importance. Compared with LR-LASSO, Random Forest showed comparable discrimination without significant reclassification improvement (NRI = −0.472; IDI = −0.067).

**Conclusions:**

A Random Forest-based ML model with an online calculator and SHAP-based interpretability supports dynamic in-hospital post-EVT mortality risk stratification, with complication predictors incorporated only after they clinically occur. Prospective external validation in geographically diverse multicenter cohorts is warranted.

## Introduction

Acute ischemic stroke caused by large vessel occlusion (AIS-LVO), defined as the acute occlusion of major intracranial arteries such as the internal carotid artery, middle cerebral artery, vertebral artery, or basilar artery, accounts for approximately 25%−40% of all ischemic strokes and is associated with disproportionately high rates of mortality and severe disability (1). Endovascular treatment (EVT) has been firmly established as the standard reperfusion strategy for eligible patients (2, 3). With continuous advances in stent retrievers and aspiration techniques, successful recanalization rates [modified Thrombolysis in Cerebral Infarction (mTICI) 2b−3] now exceed 80-90% in contemporary clinical practice (4, 5).

However, successful recanalization does not necessarily translate into favorable clinical outcomes, a phenomenon increasingly referred to as “futile recanalization” (6, 7). Despite high rates of successful recanalization, only approximately one-third of patients achieve functional independence at 90 days, even in randomized controlled trials, while a substantial proportion remain moderately to severely disabled and 15%−20% die within 90 days (6, 8). Dynamic identification of patients at high risk of post-EVT mortality during hospitalization is therefore critical for guiding post-procedural management, optimizing monitoring strategies, and supporting timely prognostic discussions with patients and their families.

Previous studies investigating EVT outcomes have predominantly focused on functional disability, most commonly assessed using the modified Rankin Scale (mRS), with relatively limited attention to mortality as a hard endpoint (9, 10). Conventional prognostic models, including SPAN-100, PRE, and HIAT2, are largely based on logistic regression and may be limited in capturing complex nonlinear relationships among clinical variables (11–13). In addition, many studies emphasize discrimination metrics while underreporting calibration, clinical utility, and internal validation, which are increasingly recommended by TRIPOD guidelines for transparent prediction model development (14).

Machine learning (ML) approaches offer the potential to model high-dimensional data and non-linear interactions, thereby improving predictive performance in stroke outcome prediction (9, 15, 16). Meanwhile, advances in explainable artificial intelligence, such as SHapley Additive exPlanations (SHAP), enable transparent interpretation of model outputs at both global and individual levels, enhancing clinical applicability (17).

In this study, we aimed to develop and internally validate a machine learning model for predicting 90-day all-cause mortality after EVT in patients with AIS-LVO, based on a real-world cohort from southwestern China, a region that remains underrepresented in current EVT prognostic evidence. We further evaluated model performance using a comprehensive framework incorporating discrimination, calibration, and clinical utility, and explored model interpretability using SHAP. Finally, we developed an accompanying online risk calculator to facilitate individualized post-EVT risk stratification and support clinical decision-making.

## Methods

### Study design and patient selection

This was a single-center retrospective cohort study for prediction model development and internal validation. We consecutively enrolled AIS-LVO patients who underwent EVT and achieved successful recanalization at the Department of Neurology, Hezhou People's Hospital, between January 2022 and May 2025. The study was conducted in accordance with the Declaration of Helsinki, and reporting follows the Transparent Reporting of a multivariable prediction model for Individual Prognosis or Diagnosis (TRIPOD) statement (18).

Inclusion criteria were: (i) age ≥18 years; (ii) baseline National Institutes of Health Stroke Scale (NIHSS) ≥6; (iii) baseline ASPECTS ≥6 (including pc-ASPECTS for posterior circulation); (iv) imaging-confirmed AIS-LVO; (v) EVT performed according to current clinical guidelines; and (vi) successful recanalization defined as mTICI 2b−3.

Exclusion criteria were: (i) pre-stroke mRS >2; (ii) intracranial hemorrhage on baseline CT; (iii) onset-to-puncture time >24 h; or (iv) loss to 90-day follow-up.

A total of 449 patients were included [288 male, 161 female; median age 68 (IQR 59–76) years]. The study was approved by the Institutional Ethics Committee of Hezhou People's Hospital (Approval No. 2024052745), and the requirement for informed consent was waived due to the retrospective design.

### Outcome definition and follow-up

The primary outcome was 90-day all-cause mortality after successful EVT. All patients were followed up at 90 days post-procedure via outpatient visits, electronic medical records, or telephone interviews. For patients who could not be reached directly, follow-up information was obtained from family members or primary caregivers. Mortality was confirmed by hospital records, death certificates, or family verification. Patients with mRS = 6 at 90 days were classified as the death group, and those with mRS < 6 constituted the survival group.

### Candidate predictor variables

Clinical data were retrospectively retrieved from electronic medical records and imaging databases. Candidate variables included: (i) demographics and medical history—sex, age, hypertension, diabetes mellitus, coronary heart disease, prior stroke, smoking history, valvular heart disease, and atrial fibrillation; (ii) admission vital signs and neurological assessment—systolic and diastolic blood pressure, NIHSS, and ASPECTS scores; (iii) laboratory parameters—measured at admission or the morning after EVT, including white blood cell count, neutrophil count, lymphocyte count, platelet count, neutrophil-to-lymphocyte ratio (NLR), platelet-to-lymphocyte ratio (PLR), D-dimer, fasting blood glucose, total cholesterol, triglycerides, and low-density lipoprotein cholesterol (LDL-C); (iv) treatment and procedural variables—intravenous thrombolysis, occlusion site, Trial of Org 10172 in Acute Stroke Treatment (TOAST) classification, onset-to-puncture time, puncture-to-recanalization time, onset-to-recanalization time, and number of thrombectomy passes; and (v) post-procedural complications—stroke-associated pneumonia, stress gastrointestinal ulcer, deep vein thrombosis, acute heart failure, acute renal insufficiency, symptomatic intracranial hemorrhage (sICH), and brain herniation.

Because several candidate predictors, including stroke-associated pneumonia, symptomatic intracranial hemorrhage, brain herniation, and acute renal insufficiency, were post-procedural in-hospital complications, the intended use of the model was dynamic post-EVT in-hospital mortality risk stratification rather than pre-procedural or immediate post-thrombectomy prognostication. These predictors were included only when clinically available during hospitalization, and model outputs were interpreted as updated mortality risk estimates as the patient's clinical status evolved.

### Data preprocessing and dataset partitioning

All analyses were performed using Python 3.11 and scikit-learn 1.4, with a fixed random seed of 42 for reproducibility. The overall proportion of missing data was low, with no candidate predictor having more than 5% missing values; therefore, all candidate predictors were retained for model development. Data preprocessing included identification of variable types, low-variance feature filtering, median imputation for continuous variables, mode imputation for categorical variables, one-hot encoding for nominal variables, and Z-score standardization for linear models. All preprocessing steps were implemented within cross-validation pipelines and fitted independently within each fold to prevent data leakage. The cohort was randomly divided into training (*n* = 359) and independent testing (*n* = 90) sets in an 8:2 ratio, stratified by 90-day mortality. The independent testing set was not used during preprocessing, hyperparameter optimization, or model selection, and was reserved only for final model evaluation.

### Model development

We constructed and compared 15 machine learning models, including linear models (logistic regression, LASSO, ElasticNet, and linear discriminant analysis), classical machine learning models (quadratic discriminant analysis, naive Bayes, support vector machine, and k-nearest neighbors), and tree-based ensemble models (decision tree, Random Forest, Extra Trees, gradient boosting, AdaBoost, XGBoost, and LightGBM). Hyperparameter optimization was performed using Bayesian optimization (30 iterations) with 5-fold stratified cross-validation, using the area under the receiver operating characteristic curve (AUC) as the optimization metric. Model selection was based on a comprehensive evaluation of discrimination, calibration, and clinical utility rather than AUC alone. The final selected model was then evaluated on the independent testing set, which had not been used during hyperparameter optimization or model selection. When candidate models showed comparable performance, the final choice prioritized calibration stability, robustness of feature-importance rankings, and interpretability in addition to discrimination; on this basis Random Forest was selected over the marginally higher cross-validation AUC of Extra Trees.

### Model evaluation

Model performance was evaluated using both probability-based and threshold-dependent metrics. Probability-based assessment included discrimination, calibration, and clinical utility. Discrimination was quantified by the area under the receiver operating characteristic curve (AUC). Calibration was assessed using calibration plots, Brier score, scaled Brier score, calibration slope, calibration-in-the-large (CITL), and the Hosmer–Lemeshow test. Given the limited number of events in the independent test set, the Hosmer–Lemeshow test was interpreted with caution. Clinical utility was evaluated using decision curve analysis (DCA), estimating net benefit across threshold probabilities of 0.10–0.60 compared with “treat-all” and “treat-none” strategies. This range was selected to reflect clinically plausible thresholds for intensified post-EVT monitoring, complication prevention, neurocritical care consultation, and prognostic communication during hospitalization. Threshold-dependent metrics included sensitivity, specificity, positive predictive value, negative predictive value, F1 score, and confusion matrix. Discrimination and classification metrics were reported with 95% confidence intervals estimated by bootstrap resampling (2,000 replicates), and precision–recall curves with average precision were additionally computed to account for class imbalance. Binary classification was initially based on a probability threshold of 0.5, with additional threshold analysis using the Youden index derived from the development set; the independent test set was not used for threshold selection. Internal validation was performed using Harrell's bootstrap optimism correction (200 iterations) for the top five models. All candidate models were subsequently evaluated in the independent test set, with final model selection based on overall performance across discrimination, calibration, and clinical utility. Model comparison was further conducted using category-free net reclassification improvement (NRI) and integrated discrimination improvement (IDI), with LR-LASSO as the reference model.

### Model interpretability and clinical translation

Model interpretability was assessed using SHAP. SHAP summary and beeswarm plots were used to quantify global feature importance and illustrate the direction of feature effects, while waterfall and force plots were applied to demonstrate individualized prediction patterns in representative cases. Permutation importance was additionally calculated to assess the robustness of feature rankings. Because post-procedural complications were included as candidate predictors, the final model was intended for post-EVT mortality risk stratification rather than early prognostication. Accordingly, model outputs were interpreted as predicted probabilities rather than strict binary classifications.

An online risk calculator was developed based on the final model to facilitate clinical application. The calculator provides individualized 90-day mortality probabilities and categorizes patients into four risk groups (low < 10%, moderate 10%−30%, high 30%−50%, and very high ≥ 50%). These categories were designed for clinical interpretation and communication and do not represent thresholds used for model evaluation.

### Statistical analysis

Statistical analyses were performed using Python 3.11 (pandas, scikit-learn, scipy, SHAP). Continuous variables were assessed for normality using the Shapiro-Wilk test; non-normally distributed data are presented as median (P25, P75) and compared using the Mann-Whitney U test. Categorical variables are presented as n (%) and compared using the chi-square test or Fisher's exact test where appropriate. All tests were two-sided, with *p* < 0.05 considered statistically significant.

## Results

### Patient characteristics

A total of 449 AIS-LVO patients meeting the inclusion criteria were enrolled. After 8:2 stratified random splitting by 90-day mortality, the training set comprised 359 patients (70 deaths, event rate 19.5%), and the independent testing set comprised 90 patients (18 deaths, event rate 20.0%). The closely matched event rates between sets confirmed the integrity of stratified sampling. Median follow-up was 90 days, and no patients were lost to follow-up after exclusion.

### Baseline comparison between death and survival groups

In the training set, the death and survival groups differed significantly across multiple variables (Table 1). Patients who died were significantly older [73.0 (67.0, 79.0) vs. 68.0 (58.0, 75.0) years; *p* = 0.001], had higher baseline NIHSS scores [17.0 (13.0, 22.0) vs. 14.0 (10.0, 18.0); *p* < 0.001], higher D-dimer levels [1.04 (0.59, 2.30) vs. 0.57 (0.41, 1.31) mg/L; *p* < 0.001], higher fasting blood glucose [6.46 (5.52, 8.45) vs. 5.64 (4.78, 6.94) mmol/L; *p* < 0.001], and a higher prevalence of atrial fibrillation (40.0% vs. 27.0%; *p* = 0.046) and prior stroke (30.0% vs. 18.0%; *p* = 0.038). Among post-procedural complications, stroke-associated pneumonia (87.1% vs. 35.3%), sICH (10.0% vs. 0.0%), brain herniation (17.1% vs. 1.0%), acute heart failure (17.1% vs. 4.5%), and acute renal insufficiency (18.6% vs. 0.3%) were all significantly more prevalent in the death group (all *p* < 0.001). Sex, hypertension, diabetes mellitus, blood pressure, ASPECTS, TOAST classification, procedural time intervals, thrombectomy passes, and lipid profiles did not differ significantly between groups (all *p* > 0.05). Similar trends were observed in the independent testing set, with age, stroke-associated pneumonia, sICH, brain herniation, and TOAST classification reaching statistical significance.

**Table 1:** Baseline characteristics of the training and testing sets stratified by 90-day mortality.

Variable	Training set (***n*** = 359)		*P*-value	Testing set (***n*** = 90)		*P*-value
	Death (*n* = 70)	Survival (*n* = 289)		Death (*n* = 18)	Survival (*n* = 72)	
Sex (Male), *n* (%)	47 (67.1)	180 (62.3)	0.536	9 (50.0)	52 (72.2)	0.128
Age, M (P25,P75), years	73.0 (67.0, 79.0)	68.0 (58.0, 75.0)	0.001	70.5 (66.0, 79.5)	62.0 (58.0, 72.3)	0.009
Hypertension, *n* (%)	43 (61.4)	175 (60.6)	1.000	8 (44.4)	37 (51.4)	0.792
Diabetes mellitus, *n* (%)	9 (12.9)	43 (14.9)	0.809	3 (16.7)	16 (22.2)	0.754
Coronary heart disease, *n* (%)	9 (12.9)	23 (8.0)	0.291	0 (0.0)	8 (11.1)	0.350
Atrial fibrillation, *n* (%)	28 (40.0)	78 (27.0)	0.046	7 (38.9)	14 (19.4)	0.117
Prior stroke, *n* (%)	21 (30.0)	52 (18.0)	0.038	3 (16.7)	11 (15.3)	1.000
Smoking history, *n* (%)	24 (34.3)	102 (35.3)	0.985	6 (33.3)	30 (41.7)	0.707
Valvular heart disease, *n* (%)	4 (5.7)	35 (12.1)	0.184	1 (5.6)	6 (8.3)	1.000
Admission SBP, M (P25,P75), mmHg	157.0 (131.3, 170.0)	150.0 (134.0, 165.0)	0.363	149.5 (137.8, 160.0)	145.0 (129.5, 165.0)	0.440
Admission DBP, M (P25,P75), mmHg	87.5 (78.0, 98.0)	87.0 (76.0, 98.0)	0.338	87.0 (76.8, 97.0)	88.5 (80.0, 95.0)	0.603
NIHSS score, M (P25,P75)	17.0 (13.0, 22.0)	14.0 (10.0, 18.0)	< 0.001	16.5 (14.3, 18.8)	13.0 (9.0, 18.0)	0.100
ASPECTS distribution, *n* (%)			0.581			0.751
6–8	19 (27.1)	67 (23.2)		5 (27.8)	15(20.8)	
9–10	51(72.9)	222 (76.8)		13 (72.2)	57 (79.2)	
WBC count, M (P25,P75), × 10^9^/L	10.30 (7.90, 12.35)	9.40 (7.40, 11.60)	0.132	9.65 (6.05, 11.78)	9.00 (7.07, 11.22)	0.976
Neutrophil count, M (P25,P75), × 10^9^/L	8.35 (6.17, 10.65)	7.40 (5.70, 9.60)	0.056	7.05 (5.10, 9.82)	7.15 (5.17, 8.93)	0.904
Lymphocyte count, M (P25,P75), × 10^9^/L	1.10 (0.90, 1.30)	1.20 (0.90, 1.70)	0.129	1.05 (0.83, 1.48)	1.15 (0.78, 1.73)	0.781
Platelet count, M (P25,P75), × 10^9^/L	227.5 (185.3, 279.0)	240.0 (200.0, 287.0)	0.176	226.0 (176.0, 253.0)	226.5 (184.8, 287.3)	0.532
NLR, M (P25,P75)	6.81 (4.87, 11.77)	6.28 (3.72, 10.38)	0.036	6.02 (2.94, 10.43)	6.39 (3.29, 10.23)	0.924
PLR, M (P25,P75)	213.6 (156.6, 290.9)	199.0 (132.6, 286.7)	0.304	202.6 (126.3, 269.8)	190.9 (126.5, 308.9)	0.552
D-dimer, M (P25,P75), mg/L	1.04 (0.59, 2.30)	0.57 (0.41, 1.31)	< 0.001	0.78 (0.58, 2.08)	0.61 (0.38, 1.22)	0.054
Fasting blood glucose, M (P25,P75), mmol/L	6.46 (5.52, 8.45)	5.64 (4.78, 6.94)	< 0.001	6.32 (5.28, 9.16)	5.71 (4.61, 7.43)	0.220
Total cholesterol, M (P25,P75), mmol/L	4.19 (3.81, 4.87)	4.32 (3.73, 4.96)	0.543	4.23 (3.53, 4.82)	4.31 (3.71, 5.05)	0.709
Triglycerides, M (P25,P75), mmol/L	0.99 (0.78, 1.31)	1.07 (0.75, 1.59)	0.554	0.96 (0.68, 1.64)	1.12 (0.86, 1.56)	0.394
LDL-C, M (P25,P75), mmol/L	2.40 (2.15, 2.83)	2.46 (2.00, 3.03)	0.753	2.50 (2.00, 2.91)	2.53 (1.96, 3.16)	0.533
Intravenous thrombolysis, *n* (%)	27 (38.6)	100 (34.6)	0.628	8 (44.4)	27 (37.5)	0.787
Occlusion site distribution, *n* (%)			< 0.001			0.815
Internal carotid artery (ICA)	7 (10.0)	57 (19.7)		4 (22.2)	12 (16.7)	
Middle cerebral artery (MCA)	26 (37.1)	160 (55.4)		10 (55.6)	40 (55.6)	
Tandem ICA–MCA occlusion	17 (24.3)	38 (13.1)		2 (11.1)	10 (13.9)	
Vertebral artery (VA)	4 (5.7)	3 (1.0)		0 (0.0)	2 (2.8)	
Basilar artery (BA)	14 (20.0)	25 (8.7)		1 (5.6)	7 (9.7)	
Tandem VA–BA occlusion	2 (2.9)	6 (2.1)		1 (5.6)	1 (1.4)	
TOAST classification, *n* (%)			0.268			0.007
LAA	30 (42.9)	155 (53.6)		5 (27.8)	47 (65.3)	
CE	26 (37.1)	86 (29.8)		7 (38.9)	18 (25.0)	
Other	14 (20.0)	48 (16.6)		6 (33.3)	7 (9.7)	
Thrombectomy passes, M (P25,P75)	2 (1, 3)	2 (1, 3)	0.067	2 (1, 3)	2 (1, 3)	0.384
Onset-to-puncture time, min	370 (207, 577)	431 (205, 729)	0.326	330 (235, 423)	444 (191, 770)	0.335
Puncture-to-recanalization time, min	51 (32, 63)	45 (35, 59)	0.280	51 (39, 64)	48 (33, 64)	0.399
Onset-to-recanalization time, min	424 (257, 633)	484 (253, 788)	0.386	378 (269, 486)	490 (236, 812)	0.343
Stroke-associated pneumonia, *n* (%)	61 (87.1)	102 (35.3)	< 0.001	12 (66.7)	16 (22.2)	< 0.001
Stress GI ulcer, *n* (%)	6 (8.6)	9 (3.1)	0.087	3 (16.7)	2 (2.8)	0.053
Deep vein thrombosis, *n* (%)	4 (5.7)	8 (2.8)	0.260	1 (5.6)	2 (2.8)	0.492
Acute heart failure, *n* (%)	12 (17.1)	13 (4.5)	< 0.001	1 (5.6)	4 (5.6)	1.000
Acute renal insufficiency, *n* (%)	13 (18.6)	1 (0.3)	< 0.001	0 (0.0)	1 (1.4)	1.000
Symptomatic intracranial hemorrhage, *n* (%)	7 (10.0)	0 (0.0)	< 0.001	3 (16.7)	1 (1.4)	0.024
Brain herniation, *n* (%)	12 (17.1)	3 (1.0)	< 0.001	3 (16.7)	0 (0.0)	0.007

Continuous variables: median (25th, 75th percentile), Mann-Whitney U test; categorical variables: n (%), chi-square or Fisher's exact test, as appropriate. M, median; P25, 25th percentile; P75, 75th percentile; NIHSS, National Institutes of Health Stroke Scale; ASPECTS, Alberta Stroke Program Early CT Score; SBP, systolic blood pressure; DBP, diastolic blood pressure; WBC, white blood cell; NLR, neutrophil-to- lymphocyte ratio; PLR, platelet-to-lymphocyte ratio; LDL-C, low-density lipoprotein cholesterol; LAA, large-artery atherosclerosis; CE, cardioembolism; GI, gastrointestinal.

### Model development and internal validation

Following Bayesian hyperparameter optimization (BayesSearchCV, 30 iterations) coupled with 5-fold stratified cross-validation, the top five models by mean cross-validation AUC were Extra Trees (CV-AUC = 0.898), Random Forest (0.877), LR-LASSO (0.866), LR-ElasticNet (0.866), and SVM (0.865), as shown in Table 2. These five models proceeded to bootstrap internal validation.

**Table 2:** Ranking of 15 machine learning models by 5-fold cross-validation AUC in the training set.

Rank	Model	5-fold CV-AUC
1	Extra Trees	0.898
2	Random Forest	0.877
3	LR-LASSO	0.866
4	LR-ElasticNet	0.866
5	SVM	0.865
6	Logistic Regression	0.862
7	Gradient Boosting	0.863
8	LDA	0.861
9	XGBoost	0.849
10	AdaBoost	0.843
11	Naive Bayes	0.831
12	LightGBM	0.817
13	Decision Tree	0.802
14	QDA	0.792
15	KNN	0.755

Models are ranked in descending order by mean cross-validation AUC across 5-fold stratified cross-validation in the training set (n = 359).

**Figure 1:**
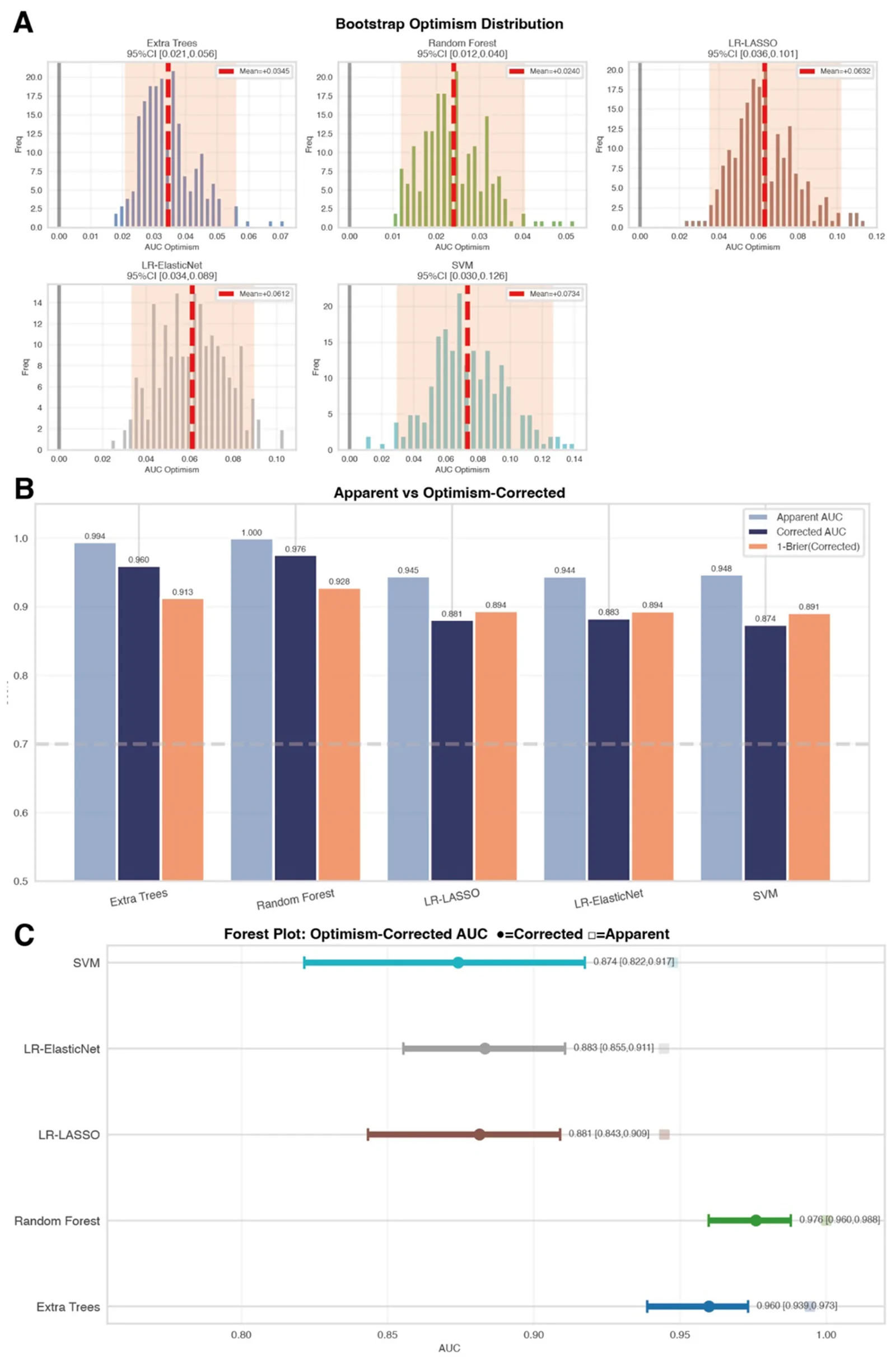
Bootstrap internal validation of the five leading models. Bootstrap optimism correction was performed for the five models with the highest cross-validation AUCs. **(A)** Distribution of AUC optimism across 200 bootstrap resamples. The dashed line indicates mean optimism, and the shaded area represents the 95% confidence interval. **(B)** Comparison of apparent and optimism-corrected AUCs. **(C)** Forest plot of optimism-corrected AUCs with 95% confidence intervals. Although Random Forest and Extra Trees showed the highest corrected AUCs, their calibration slopes indicated potential overfitting in the development set.

The top five models underwent 200-iteration bootstrap optimism correction (Table 3, Figure 1). Apparent AUCs were uniformly high (0.945–1.000), with optimism-corrected AUCs remaining within 0.874–0.976. Random Forest had an apparent AUC of 1.000 with optimism of +0.024, yielding a corrected AUC of 0.976 (95% CI: 0.960–0.988); Extra Trees had a corrected AUC of 0.960 (95% CI: 0.939–0.973). Notably, both Random Forest and Extra Trees showed substantially deviant calibration slopes on the training set (9.48 and −10.96, respectively), suggesting overfitting, whereas LR-LASSO, LR-ElasticNet, and SVM showed corrected slopes (0.65, 0.73, 0.85) closer to the ideal value of 1.

**Table 3:** Bootstrap optimism-corrected internal validation results for the top five models (200 iterations).

Model	Apparent AUC	Corrected AUC	Optimism	Apparent Brier	Corrected Brier	Apparent slope	Corrected slope
Random Forest	1.000	0.976	+0.024	0.0305	0.0718	15.56	9.48
Extra Trees	0.994	0.960	+0.035	0.0572	0.0869	5.72	−10.96
LR-LASSO	0.945	0.881	+0.063	0.0733	0.1060	1.58	0.65
LR-ElasticNet	0.944	0.883	+0.061	0.0744	0.1063	1.65	0.73
SVM	0.948	0.874	+0.073	0.0806	0.1088	2.69	0.85

Internal validation was performed using Harrell's bootstrap optimism correction with 200 iterations on the top five models. Optimism (apparent – bootstrap-validated performance) was calculated for each metric and subtracted from the apparent value to obtain the optimism-corrected estimate. Calibration slopes substantially deviating from 1.0 indicate potential overfitting.

### Independent test-set performance and clinical utility

**Figure 2:**
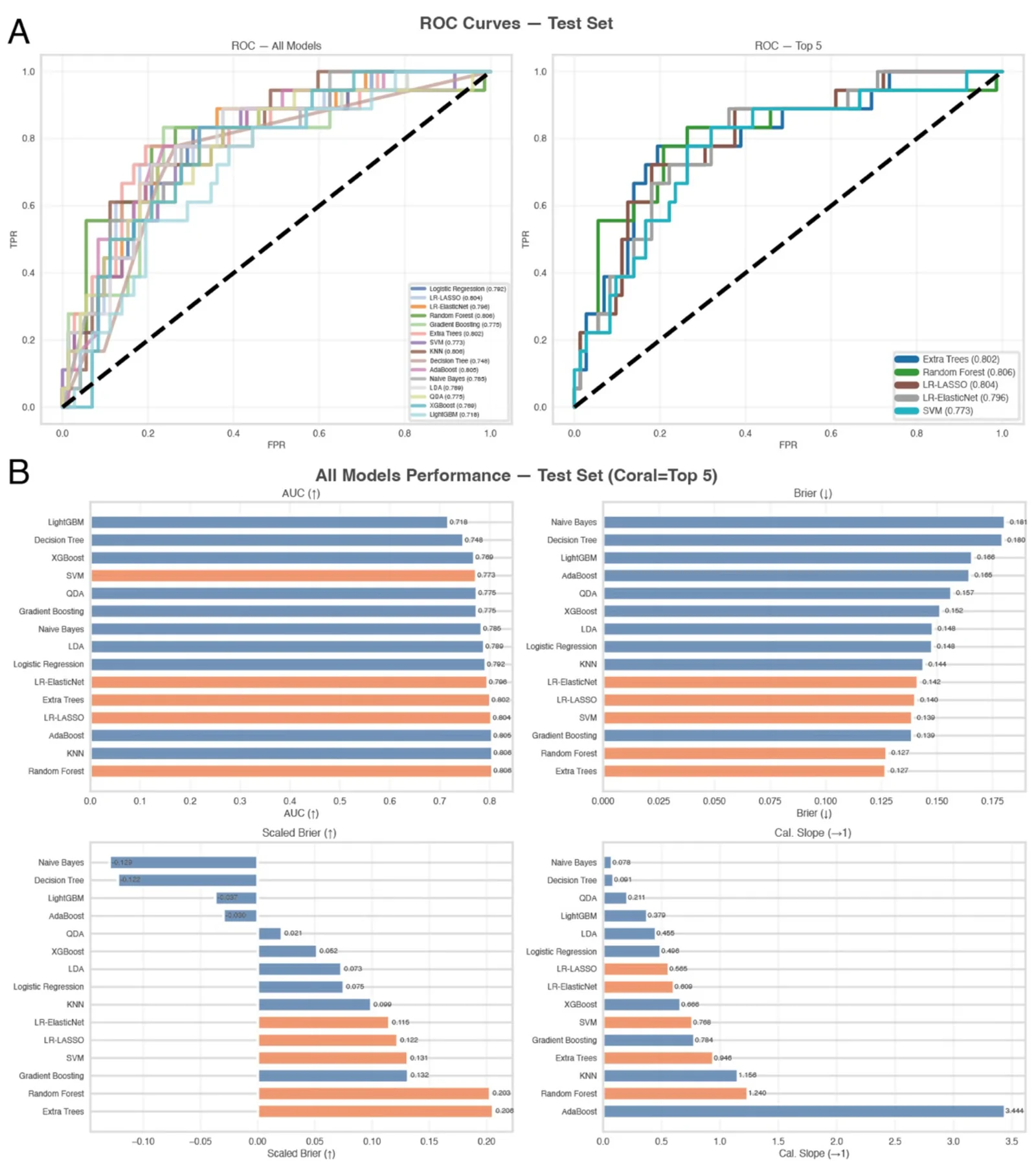
Discrimination performance and multidimensional model comparison in the independent test set. **(A)** Receiver operating characteristic curves of all 15 candidate models and the five leading models in the independent test set. AUC values are shown in parentheses. **(B)** Multidimensional comparison of model performance, including discrimination, Brier score, scaled Brier score, and calibration slope. Random Forest achieved the best overall balance between discrimination and calibration and was selected as the final model.

**Figure 3:**
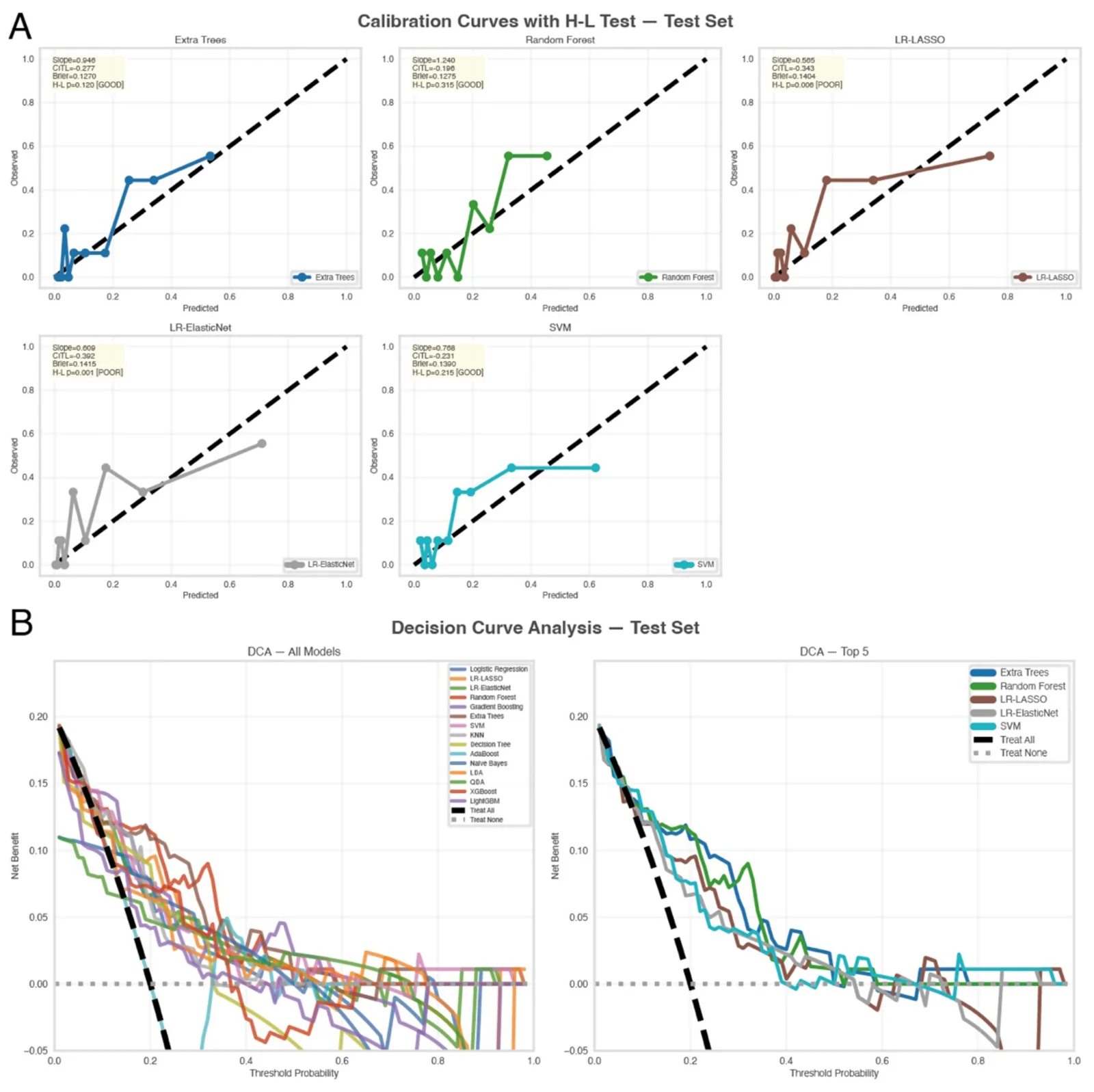
Calibration and clinical utility of candidate models in the independent test set. **(A)** Calibration curves of the five leading models. The dashed diagonal line represents perfect calibration. Model-specific calibration slope, calibration-in-the-large, Brier score, and Hosmer–Lemeshow *p* value are shown. **(B)** Decision curve analysis of all models and the five leading models. The dashed and dotted reference lines represent “treat-all” and “treat-none” strategies, respectively. Curves above both reference strategies indicate positive clinical net benefit across the corresponding threshold probability range.

Performance of all 15 models on the independent test set (*n* = 90) is shown in Table 4. Corresponding 95% confidence intervals for AUC, sensitivity, specificity, and Brier score are provided in Supplementary Table S1. The top five models ranked by test-set AUC were Random Forest (0.806), KNN (0.806), AdaBoost (0.805), LR-LASSO (0.804), and Extra Trees (0.802); ROC curves and overall multidimensional comparison are shown in Figure 2. Calibration assessment by the Hosmer–Lemeshow test (Figure 3A) was performed on the five models selected via cross-validation AUC ranking (Table 3): Random Forest (χ^2^ = 9.34, *p* = 0.315), Extra Trees (*p* = 0.120), and SVM (*p* = 0.215) demonstrated adequate calibration, whereas LR-LASSO (*p* = 0.006) and LR-ElasticNet (*p* = 0.001) showed inadequate calibration. Decision curve analysis (Figure 3B) showed that the top five models provided substantially greater net benefit than “treat-all” or “treat-none” strategies across threshold probabilities of approximately 0.10–0.60, with Random Forest and Extra Trees yielding the highest net benefit at threshold probabilities of 0.20–0.40. Considering both discrimination (AUC) and calibration (Hosmer–Lemeshow test, Brier score, calibration slope), Random Forest was selected as the final model based on its overall balance of discrimination, calibration, and clinical utility, with an AUC of 0.806, Brier score of 0.128, calibration slope of 1.24, and Hosmer–Lemeshow *p* = 0.315. Threshold-dependent classification metrics and confusion matrices at the 0.5 probability threshold are summarized for all models in Supplementary Table S2 and illustrated for the leading models in Supplementary Figure S2. Given the class imbalance, precision–recall performance was additionally examined, with average precision values reported in Supplementary Table S3 and precision–recall curves in Supplementary Figure S1.

**Table 4:** Performance of all 15 machine learning models on the independent test set (*n* = 90).

Model	AUC	Sensitivity	Specificity	PPV	F1	Brier	Cal. Slope	H-L *p* value
**Random Forest**	0.806	0.111	0.986	0.667	0.190	0.128	1.240	0.315
KNN	0.806	0.000	1.000	0.000	0.000	0.144	1.156	—
AdaBoost	0.805	0.111	0.972	0.500	0.182	0.165	3.444	—
**LR-LASSO**	0.804	0.278	0.944	0.556	0.370	0.140	0.565	0.006
**Extra Trees**	0.802	0.111	0.972	0.500	0.182	0.127	0.946	0.120
**LR-ElasticNet**	0.796	0.278	0.944	0.556	0.370	0.142	0.609	0.001
Logistic Regression	0.792	0.278	0.944	0.556	0.370	0.148	0.496	—
LDA	0.789	0.278	0.944	0.556	0.370	0.148	0.455	—
Naive Bayes	0.785	0.389	0.903	0.500	0.438	0.181	0.078	—
Gradient Boosting	0.775	0.167	0.986	0.750	0.273	0.139	0.784	—
**QDA**	0.775	0.167	0.986	0.750	0.273	0.157	0.211	—
**SVM**	0.773	0.222	0.944	0.500	0.308	0.139	0.768	0.215
XGBoost	0.769	0.000	0.958	0.000	0.000	0.152	0.666	—
Decision Tree	0.748	0.167	0.903	0.300	0.214	0.180	0.091	—
LightGBM	0.718	0.111	0.958	0.400	0.174	0.166	0.379	—

“—”, Hosmer–Lemeshow test not performed (computed only for the five models selected by cross-validation AUC); calibration slope was computed for all models. Corresponding 95% confidence intervals are provided in Supplementary Table S1. PPV, positive predictive value; Cal. slope, calibration slope; H-L, Hosmer–Lemeshow.

### Reclassification analysis

**Figure 4:**
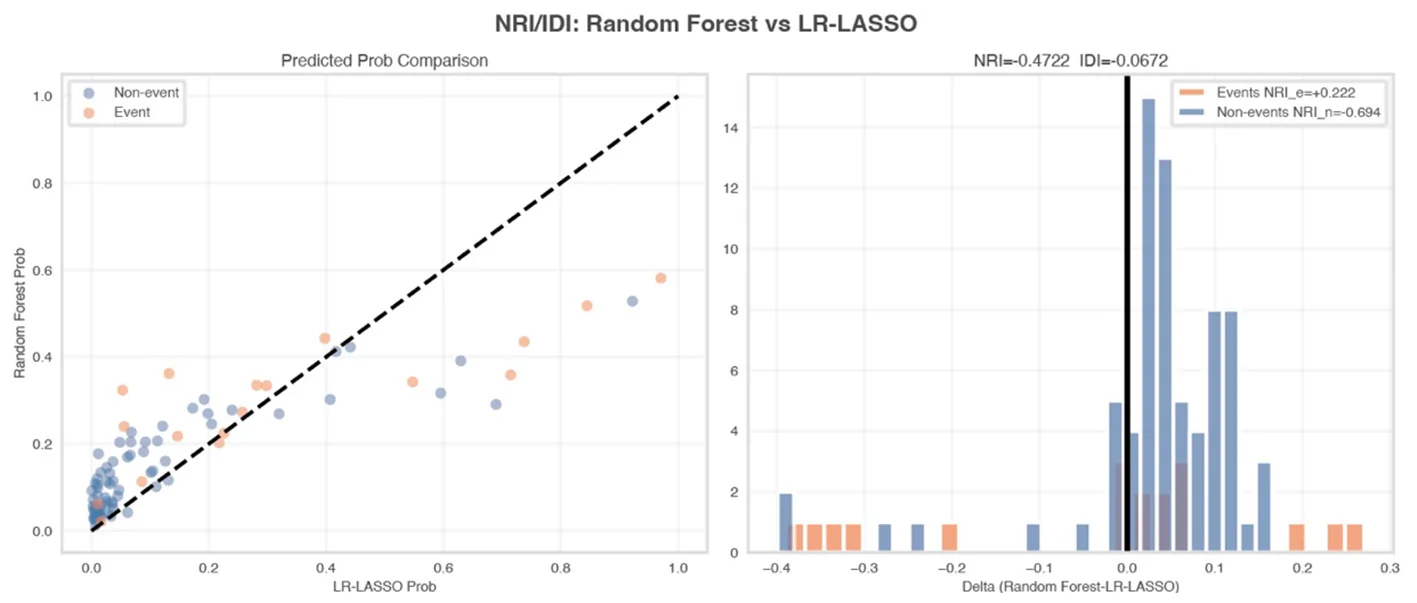
Reclassification performance of Random Forest compared with LR-LASSO. The reclassification performance of the final Random Forest model was compared with LR-LASSO as the reference model. **(A)** Scatter plot of predicted probabilities from Random Forest and LR-LASSO. Each point represents one patient; colors indicate observed 90-day mortality status. The diagonal line indicates equal predicted probabilities between models. **(B)** Distribution of probability differences between Random Forest and LR-LASSO. Random Forest did not provide a meaningful overall reclassification improvement compared with LR-LASSO.

Compared with LR-LASSO as the reference model, Random Forest demonstrated similar discrimination but did not yield significant net reclassification improvement on the independent test set (Figure 4). NRI was −0.472 (event NRI = +0.222; non-event NRI = −0.694), and IDI was −0.067 (event IDI = −0.034; non-event IDI = +0.033). These results indicate that Random Forest assigned higher predicted probabilities than LR-LASSO in the event group (deceased patients), thereby identifying additional high-risk individuals, but tended to overestimate risk in survivors, resulting in no overall reclassification benefit. Considering discrimination, calibration, and reclassification together, Random Forest was selected as the final predictive model.

### Clinical interpretability of the final model

**Figure 5:**
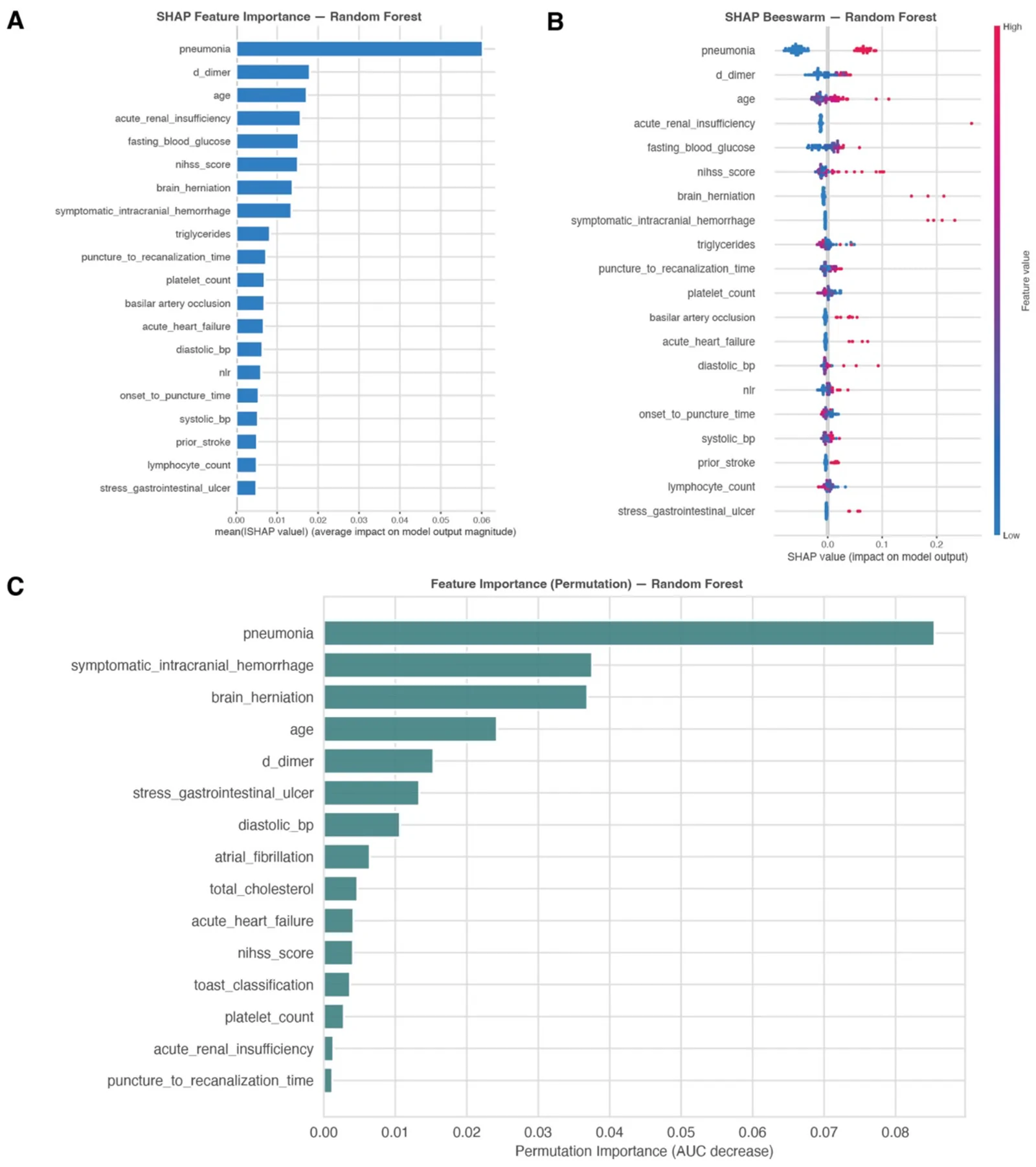
Global interpretability of the final Random Forest model. SHAP and permutation importance analyses were used to interpret the final Random Forest model. **(A)** SHAP bar plot showing the mean absolute SHAP value of each predictor. **(B)** SHAP beeswarm plot showing the direction and magnitude of each predictor's contribution to individual predictions. Red indicates higher feature values and blue indicates lower feature values. **(C)** Permutation importance based on the decrease in AUC after feature perturbation. Stroke-associated pneumonia, D-dimer, age, acute renal insufficiency, fasting blood glucose, NIHSS score, brain herniation, and symptomatic intracranial hemorrhage were the dominant contributors to predicted mortality risk.

SHAP analysis of the optimal Random Forest model on the independent test set is presented in Figure 5. The SHAP bar plot (Figure 5A) ranked the top 10 features as: stroke-associated pneumonia, D-dimer, age, acute renal insufficiency, fasting blood glucose, NIHSS score, brain herniation, sICH, triglycerides, and puncture-to-recanalization time. Stroke-associated pneumonia exhibited the highest mean (|SHAP|), substantially exceeding all other features. The SHAP beeswarm plot (Figure 5B) confirmed the directional relationships: the presence of stroke-associated pneumonia, brain herniation, sICH, acute renal insufficiency, and acute heart failure (red points on the right) substantially increased predicted death risk; higher D-dimer, fasting blood glucose, NIHSS, and older age also pushed predictions toward higher risk—all consistent with established clinical understanding. Permutation importance showed a consistent feature ranking, supporting the robustness of the SHAP findings (Figure 5C).

**Figure 6:**
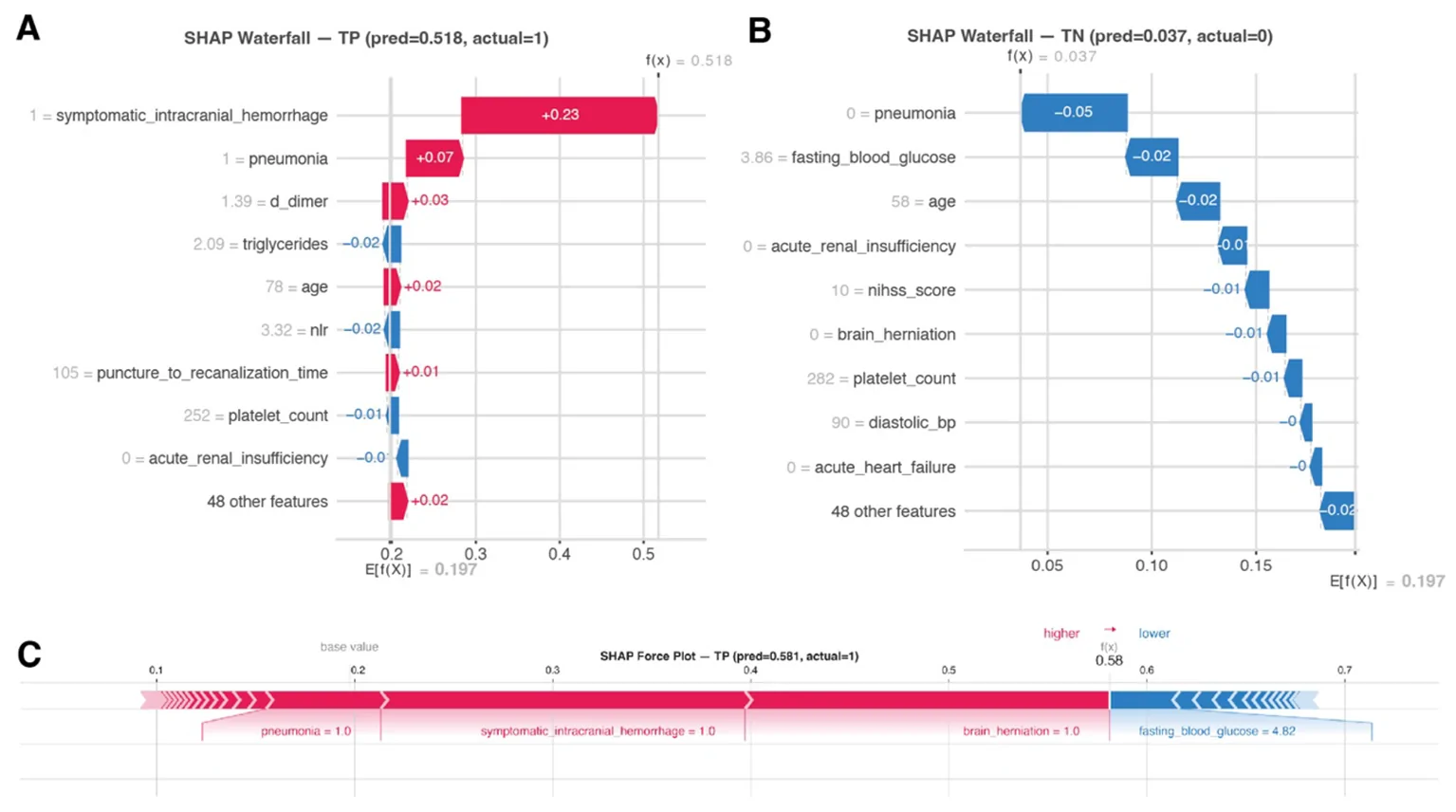
Individualized SHAP explanations for representative patients. Individual-level SHAP plots illustrate how the Random Forest model generated patient-specific mortality probability estimates. **(A)** SHAP waterfall plot for a true-positive case with observed 90-day mortality. **(B)** SHAP waterfall plot for a true-negative case with observed survival. **(C)** SHAP force plot for the true-positive case. Red features increased the predicted mortality probability, whereas blue features decreased it. The baseline value represents the average model prediction before incorporating patient-specific features.

Individual-level SHAP explanations further illustrated how the model combined patient-specific risk factors to generate mortality probability estimates (Figure 6). In the representative true-positive case, the predicted high mortality risk was mainly driven by symptomatic intracranial hemorrhage, stroke-associated pneumonia, elevated D-dimer, and advanced age. Conversely, the representative true-negative case showed a low predicted mortality risk due to absence of major complications, younger age, lower glucose level, and lower neurological severity.

### Online risk calculator

Based on the optimal Random Forest model, we developed a publicly accessible online risk calculator (https://istroke.cn). Clinicians input the top 10 SHAP-ranked features—age, NIHSS score, D-dimer, fasting blood glucose, triglycerides, puncture-to-recanalization time, and the binary status of stroke-associated pneumonia, sICH, brain herniation, and acute renal insufficiency—to obtain individualized 90-day mortality probability estimates with risk stratification (low: < 10%, moderate: 10%−30%, high: 30%−50%, very high: ≥ 50%) and corresponding clinical recommendations.

## Discussion

In this single-center retrospective cohort of 449 patients with AIS-LVO who underwent successful EVT, we systematically developed and internally validated 15 ML models for predicting 90-day all-cause mortality. The Random Forest model demonstrated the best overall performance on the independent test set (AUC = 0.806; Hosmer–Lemeshow *p* = 0.315, indicating adequate calibration). SHAP and permutation importance analyses concordantly identified stroke-associated pneumonia, D-dimer, age, acute renal insufficiency, fasting blood glucose, NIHSS score, brain herniation, and symptomatic intracranial hemorrhage as the most influential predictors. Our study extends the existing literature in three key ways: (i) we focused on the hard endpoint of 90-day mortality rather than functional disability; (ii) we adopted a multidimensional evaluation framework covering discrimination, calibration, optimism, clinical utility, and individualized interpretability, in line with the TRIPOD statement (18); and (iii) we developed an accessible online calculator with risk stratification to facilitate bedside translation.

Several prior studies have applied ML to predict outcomes after EVT for AIS-LVO. Nishi et al. applied random forest and other ML algorithms to standard pretreatment clinical variables in 387 EVT patients (with external validation in 115), achieving an external validation AUC of 0.87 for 90-day functional outcome and significantly outperforming five established pretreatment risk scores (SPAN-Index, PRE, THRIVE, HIAT, HIAT2) (19). Subsequently, Brugnara et al. used gradient boosting classifiers integrating clinical, multimodal imaging, and angiographic features in 246 patients to predict 90-day functional outcome (mRS-90), achieving an AUC of 0.856 (95% CI, 0.850–0.861) when post-procedural variables were included, with 24-h NIHSS, premorbid mRS, and final infarction volume identified as the most important predictors (9). Li et al. (20) further combined DWI-based radiomics features with a support vector machine classifier in 260 EVT patients, extracting 1,936 radiomic features and selecting six for the final model, which achieved a test-set AUC of 0.920 for predicting post-thrombectomy prognosis. Most recently, von Braun et al. developed a deep learning framework trained on 405 EVT patients (304 for training, 50 for internal testing, and 51 for external testing) that simulated patient-specific tissue and clinical outcomes under both successful and unsuccessful reperfusion scenarios, achieving a Dice score of 0.52 for tissue outcome prediction and a median absolute error of 3.0 NIHSS points for discharge NIHSS prediction on external validation, outperforming both threshold-based clinical methods and generalized linear models (21). Our Random Forest model achieved a test-set AUC of 0.806—within the range of these prior reports. While imaging- or deep learning–based approaches achieve higher discrimination, our model prioritizes interpretability and deployability using routinely available clinical variables. More importantly, our framework adds rigorous calibration assessment (Hosmer–Lemeshow *p* = 0.315; Brier score = 0.128), bootstrap optimism correction, and decision curve analysis—components frequently omitted from the existing ML stroke prediction literature (18).

Among the seven most influential predictors identified by SHAP and permutation importance, stroke-associated pneumonia emerged as the dominant feature, with mean (|SHAP|) and AUC-decrease values both substantially exceeding those of other features. This observation aligns with extensive evidence linking stroke-associated pneumonia to a 2- to 3-fold increase in short-term mortality and substantially worse 90-day outcomes (22, 23). The proposed mechanism involves the “stroke-induced immunodepression syndrome,” in which acute brain injury triggers sympathetic and hypothalamic–pituitary–adrenal axis activation, leading to systemic immune suppression that, combined with dysphagia-induced aspiration, markedly increases vulnerability to pulmonary infections (24, 25). The pronounced between-group contrast observed in our cohort (87.1% vs. 35.3%) likely reflects the high baseline severity of our population (median NIHSS score of 17). Malignant cerebral edema with subsequent herniation and symptomatic intracranial hemorrhage are recognized as the two leading causes of death after EVT, accounting for approximately 50% and 13% of post-EVT deaths, respectively (26). In line with this, our death group showed significantly higher rates of both events (sICH: 10.0% vs. 0.0%; herniation: 17.1% vs. 1.0%; both *p* < 0.001). As discussed previously, however, these complications likely lie on the causal pathway to mortality and should be regarded as proximal markers of severity rather than independent risk factors.

The remaining top predictors—age, baseline NIHSS score, D-dimer, and fasting blood glucose—are well-established prognostic factors with strong biological plausibility, and their inclusion in our model is consistent with prior literature. Age and NIHSS reflect baseline cerebral reserve and stroke severity, respectively, and feature prominently in classical predictive scores including SPAN-100, PRE, and HIAT2 (11–13). Notably, the HERMES meta-analysis demonstrated that EVT remained beneficial even in patients aged ≥ 80 years (6), suggesting that mortality risk in elderly patients is multifactorial rather than attributable to age *per se*. Elevated D-dimer indicates a high thrombotic burden and has been identified as an independent predictor of poor outcome in acute ischemic stroke (27). Hyperglycemia, in turn, has been associated with infarct extension, hemorrhagic transformation, and blood–brain barrier disruption, and has been linked to worse functional outcomes and higher rates of symptomatic intracranial hemorrhage in patients with large ischemic-region strokes (28). Both markers were significantly elevated in our death group (D-dimer: 1.04 vs. 0.57 mg/L; fasting glucose: 6.46 vs. 5.64 mmol/L; both *p* < 0.001).

Decision curve analysis demonstrated that the top five models provided meaningful net benefit across threshold probabilities of 0.10–0.60, a range selected to approximate clinically realistic decision thresholds for dynamic in-hospital post-EVT management. At lower thresholds, the model may flag moderate-risk patients warranting enhanced monitoring and complication prophylaxis, whereas at higher thresholds it may identify very-high-risk patients who could benefit from neurocritical care, aggressive complication prevention, and early family communication regarding goals of care. Combined with SHAP-based individualized explanations and an accessible online calculator, the model may serve as a useful adjunct to clinical judgment, supporting standardized risk-based decision-making across centers with varying expertise. Notably, although Random Forest demonstrated a marginally higher AUC than LR-LASSO (0.806 vs. 0.804), the negative NRI (−0.472) and IDI (−0.067) indicated that any advantage in event-group probability assignment was offset by overestimation in survivors—consistent with the systematic review by Christodoulou et al., which concluded that ML models do not consistently outperform logistic regression in modestly sized datasets with low event counts (29). The pronounced overfitting observed in Random Forest and Extra Trees (calibration slopes of 9.48 and −10.96; apparent training AUC of 1.000 dropping to 0.806 on the independent test set) underscores the value of our integrated evaluation framework combining Bayesian hyperparameter optimization, bootstrap optimism correction, independent test-set validation, and multidimensional assessment (DCA, NRI/IDI).

This study has several limitations. First, this single-center retrospective study had a relatively modest sample size (*N* = 449; 88 events) and a small independent test set (*n* = 90; 18 events), which may increase the risk of selection bias, constrain generalizability and widen the confidence intervals around performance estimates. The cohort was drawn from a nationally certified Advanced Stroke Center in Guangxi, southwestern China, a region underrepresented in EVT prognostic research and with cerebrovascular risk factor profiles distinct from those of northern Chinese and Western populations; therefore, the findings may reflect a specific geographic, population, healthcare-system, and practice-pattern context. Although bootstrap optimism correction and an independent held-out test set were applied during internal validation, the model has not yet undergone temporal validation or external validation in geographically independent cohorts. Prospective multicenter validation in temporally and geographically distinct populations remains essential before broader clinical implementation. Second, several influential predictors, including stroke-associated pneumonia, symptomatic intracranial hemorrhage, brain herniation, and acute renal insufficiency, were post-procedural complications rather than baseline variables. These variables may lie on the causal pathway between severe stroke, post-EVT complications, and subsequent mortality, and their inclusion may introduce potential temporal leakage if the model is interpreted as a pre-procedural or immediate post-thrombectomy prognostic tool. Accordingly, the present model should be regarded not as a baseline-only or immediate post-thrombectomy prediction tool, but as a dynamic in-hospital post-EVT risk-stratification model intended to be updated as the patient's clinical status evolves during hospitalization. Such dynamic evaluation is particularly relevant in the Chinese clinical context, where post-procedural management decisions—including escalation to neurocritical care, intensification of complication surveillance, and structured family communication regarding goals of care—carry substantial weight for both clinicians and families. By integrating evolving clinical and complication data, the model may support shared decision-making and individualized care planning at the bedside, complementing rather than replacing clinical judgment. In addition, the model was not directly compared with established mortality or prognostic scores after EVT. Future studies should evaluate whether this ML-based model provides incremental clinical value beyond conventional risk scores and simpler interpretable models. Finally, although decision curve analysis and SHAP-based explanations supported the model's potential clinical utility and interpretability, these findings should be regarded as hypothesis-generating. The accompanying online calculator has not yet been prospectively validated, and its real-world impact on clinical decision-making remains to be established.

## Conclusions

We developed and internally validated a Random Forest-based machine learning model for predicting 90-day all-cause mortality after EVT in patients with acute ischemic stroke due to large vessel occlusion. The model demonstrated good discrimination (test-set AUC = 0.806), satisfactory calibration (Hosmer–Lemeshow *p* = 0.315), and favorable clinical net benefit. Stroke-associated pneumonia, symptomatic intracranial hemorrhage, brain herniation, age, D-dimer, fasting blood glucose, and the NIHSS score were the most influential predictors. The accompanying online calculator provides a preliminary and interpretable tool for individualized dynamic in-hospital mortality risk stratification after EVT, but prospective external validation in geographically and temporally independent multicenter cohorts is required before clinical implementation.

## Data Availability

The datasets presented in this article are not readily available because the datasets generated and/or analyzed during the current study are not publicly available due to institutional data protection regulations. Data access is subject to approval by the relevant institutional authority and may be obtained from the corresponding author upon reasonable request and with permission from the institution. Requests to access the datasets should be directed to scilb1015@126.com.
