# Supplementary material for: Interpretable machine learning–based prediction of 90-day mortality after endovascular treatment in acute large vessel occlusion stroke
Source: Front Neurol. 2026 Sep 3;17:1878562. doi: 10.3389/fneur.2026.1878562 (PMC13581525; doi:10.3389/fneur.2026.1878562)
Supplement: Supplementary file 1 [file Supplementary_File_1.docx]

**Supplementary Material**

**Interpretable machine learning–based prediction of 90-day mortality after endovascular treatment in acute large vessel occlusion stroke**

Yuanfang Zhou, Xiaoxia Li, Guokuan Feng, Chuanhang Lin, Bao Liao*

** Correspondence: Bao Liao, scilb1015@126.com*

This Supplementary Material expands the independent test-set evaluation reported in the main text (Table 4; Figures 2–3). All analyses were performed on the independent test set (n = 90; 18 deaths) using the models described in the Methods. Ninety-five percent confidence intervals were obtained by bootstrap resampling (2000 replicates), and models are ordered by test-set AUC, consistent with Table 4. In keeping with the intended use of the model as a dynamic in-hospital post-EVT mortality risk-stratification tool, probability-based risk estimates rather than fixed binary classifications are emphasized; threshold-dependent metrics at the default 0.5 cut-off are reported for completeness and, given the low event rate, show the expected low sensitivity, which should be interpreted accordingly.

**1. Supplementary Tables**

**Supplementary Table S1. Discrimination and classification performance with 95% confidence intervals for all 15 machine-learning models in the independent test set (n = 90).**

| **Model** | **AUC (95% CI)** | **Sensitivity (95% CI)** | **Specificity (95% CI)** | **Brier score (95% CI)** |
| --- | --- | --- | --- | --- |
| **Random Forest** | 0.806 (0.654–0.924) | 0.111 (0.000–0.278) | 0.986 (0.949–1.000) | 0.128 (0.083–0.176) |
| **KNN** | 0.806 (0.694–0.903) | 0.000 (0.000–0.000) | 1.000 (1.000–1.000) | 0.144 (0.095–0.199) |
| **AdaBoost** | 0.805 (0.687–0.901) | 0.111 (0.000–0.278) | 0.972 (0.928–1.000) | 0.165 (0.143–0.187) |
| **LR-LASSO** | 0.804 (0.684–0.902) | 0.278 (0.077–0.526) | 0.944 (0.885–0.987) | 0.140 (0.088–0.197) |
| **Extra Trees** | 0.802 (0.674–0.900) | 0.111 (0.000–0.273) | 0.972 (0.930–1.000) | 0.127 (0.082–0.179) |
| **LR-ElasticNet** | 0.796 (0.681–0.903) | 0.278 (0.087–0.500) | 0.944 (0.886–0.986) | 0.142 (0.089–0.200) |
| **Logistic Regression** | 0.792 (0.681–0.899) | 0.278 (0.071–0.500) | 0.944 (0.889–0.987) | 0.148 (0.090–0.207) |
| **LDA** | 0.789 (0.655–0.894) | 0.278 (0.067–0.500) | 0.944 (0.885–0.987) | 0.148 (0.091–0.213) |
| **Naive Bayes** | 0.785 (0.658–0.888) | 0.389 (0.167–0.625) | 0.903 (0.829–0.970) | 0.181 (0.104–0.257) |
| **Gradient Boosting** | 0.775 (0.635–0.885) | 0.167 (0.000–0.364) | 0.986 (0.955–1.000) | 0.139 (0.087–0.196) |
| **QDA** | 0.775 (0.640–0.887) | 0.167 (0.000–0.375) | 0.986 (0.956–1.000) | 0.157 (0.093–0.228) |
| **SVM** | 0.773 (0.641–0.889) | 0.222 (0.050–0.445) | 0.944 (0.892–0.987) | 0.139 (0.087–0.187) |
| **XGBoost** | 0.769 (0.660–0.875) | 0.000 (0.000–0.000) | 0.958 (0.911–1.000) | 0.152 (0.096–0.209) |
| **Decision Tree** | 0.748 (0.632–0.860) | 0.167 (0.000–0.357) | 0.903 (0.836–0.961) | 0.180 (0.120–0.243) |
| **LightGBM** | 0.718 (0.591–0.842) | 0.111 (0.000–0.278) | 0.958 (0.905–1.000) | 0.166 (0.105–0.234) |

*Values are point estimates with 95% confidence intervals (in parentheses) from 2000 bootstrap resamples of the independent test set. AUC, area under the receiver operating characteristic curve; CI, confidence interval; LR, logistic regression; LDA, linear discriminant analysis; QDA, quadratic discriminant analysis; SVM, support vector machine; KNN, k-nearest neighbors.*

**Supplementary Table S2. Confusion matrices and threshold-dependent classification metrics at the default 0.5 probability threshold for all 15 models in the independent test set (n = 90).**

| **Model** | **TN** | **FP** | **FN** | **TP** | **Sens.** | **Spec.** | **PPV** | **NPV** | **Acc.** | **F1** | **AUC** |
| --- | --- | --- | --- | --- | --- | --- | --- | --- | --- | --- | --- |
| **Random Forest** | 71 | 1 | 16 | 2 | 0.111 | 0.986 | 0.667 | 0.816 | 0.811 | 0.190 | 0.806 |
| **KNN** | 72 | 0 | 18 | 0 | 0.000 | 1.000 | — | 0.800 | 0.800 | — | 0.806 |
| **AdaBoost** | 70 | 2 | 16 | 2 | 0.111 | 0.972 | 0.500 | 0.814 | 0.800 | 0.182 | 0.805 |
| **LR-LASSO** | 68 | 4 | 13 | 5 | 0.278 | 0.944 | 0.556 | 0.840 | 0.811 | 0.370 | 0.804 |
| **Extra Trees** | 70 | 2 | 16 | 2 | 0.111 | 0.972 | 0.500 | 0.814 | 0.800 | 0.182 | 0.802 |
| **LR-ElasticNet** | 68 | 4 | 13 | 5 | 0.278 | 0.944 | 0.556 | 0.840 | 0.811 | 0.370 | 0.796 |
| **Logistic Regression** | 68 | 4 | 13 | 5 | 0.278 | 0.944 | 0.556 | 0.840 | 0.811 | 0.370 | 0.792 |
| **LDA** | 68 | 4 | 13 | 5 | 0.278 | 0.944 | 0.556 | 0.840 | 0.811 | 0.370 | 0.789 |
| **Naive Bayes** | 65 | 7 | 11 | 7 | 0.389 | 0.903 | 0.500 | 0.855 | 0.800 | 0.438 | 0.785 |
| **Gradient Boosting** | 71 | 1 | 15 | 3 | 0.167 | 0.986 | 0.750 | 0.826 | 0.822 | 0.273 | 0.775 |
| **QDA** | 71 | 1 | 15 | 3 | 0.167 | 0.986 | 0.750 | 0.826 | 0.822 | 0.273 | 0.775 |
| **SVM** | 68 | 4 | 14 | 4 | 0.222 | 0.944 | 0.500 | 0.829 | 0.800 | 0.308 | 0.773 |
| **XGBoost** | 69 | 3 | 18 | 0 | 0.000 | 0.958 | 0.000 | 0.793 | 0.767 | — | 0.769 |
| **Decision Tree** | 65 | 7 | 15 | 3 | 0.167 | 0.903 | 0.300 | 0.813 | 0.756 | 0.214 | 0.748 |
| **LightGBM** | 69 | 3 | 16 | 2 | 0.111 | 0.958 | 0.400 | 0.812 | 0.789 | 0.174 | 0.718 |

*Metrics are computed at the default 0.5 probability threshold. TN, true negative; FP, false positive; FN, false negative; TP, true positive; Sens., sensitivity; Spec., specificity; PPV, positive predictive value; NPV, negative predictive value; Acc., accuracy; F1, F1 score; AUC, area under the ROC curve. Dashes denote metrics that are undefined when a model makes no positive predictions. Low sensitivity at this threshold reflects the low 90-day mortality rate (20% in the test set); probability-based risk stratification (calibration and decision-curve analysis; main text) is therefore emphasized over fixed-threshold classification.*

**Supplementary Table S3. Average precision and AUC for all 15 models in the independent test set (n = 90).**

| **Model** | **Average precision** | **AUC** |
| --- | --- | --- |
| **Random Forest** | 0.569 | 0.806 |
| **KNN** | 0.480 | 0.806 |
| **AdaBoost** | 0.470 | 0.805 |
| **LR-LASSO** | 0.524 | 0.804 |
| **Extra Trees** | 0.528 | 0.802 |
| **LR-ElasticNet** | 0.506 | 0.796 |
| **Logistic Regression** | 0.509 | 0.792 |
| **LDA** | 0.509 | 0.789 |
| **Naive Bayes** | 0.459 | 0.785 |
| **Gradient Boosting** | 0.480 | 0.775 |
| **QDA** | 0.501 | 0.775 |
| **SVM** | 0.504 | 0.773 |
| **XGBoost** | 0.390 | 0.769 |
| **Decision Tree** | 0.367 | 0.748 |
| **LightGBM** | 0.356 | 0.718 |

*AP, average precision (area under the precision–recall curve); AUC, area under the receiver operating characteristic curve. The no-skill baseline equals the test-set event rate (0.200). Values correspond to the precision–recall curves in Supplementary Figure S1.*

**2. Supplementary Figures**

**Supplementary Figure S1. Precision–recall curves in the independent test set (n = 90).**

| **(A) All 15 candidate models**   | **(B) Five leading models**   |
| --- | --- |
| **(C) Final Random Forest model**   |  |

*(A) All 15 candidate models; (B) the five models with the highest test-set AUC (Random Forest, k-nearest neighbors, AdaBoost, LR-LASSO, and Extra Trees); (C) the final Random Forest model (average precision = 0.569). The dashed line indicates the no-skill baseline equal to the event rate (0.200). Precision–recall curves are presented because they are more informative than ROC curves under class imbalance.*

**Supplementary Figure S2. Confusion matrices at the default 0.5 probability threshold for the five leading models in the independent test set (n = 90).**

| **(A) Random Forest (final model)**   | **(B) LR-LASSO**   |
| --- | --- |
| **(C) Extra Trees**   | **(D) k-nearest neighbors**   |
| **(E) AdaBoost**   |  |

*Rows correspond to the observed class and columns to the predicted class. Low sensitivity at this threshold reflects the low event rate and is consistent with the emphasis on probabilistic risk stratification described in the main text.*
